# Does online masked priming pass the test? The effects of prime exposure duration on masked identity priming

**DOI:** 10.3758/s13428-021-01742-y

**Published:** 2022-03-16

**Authors:** Bernhard Angele, Ana Baciero, Pablo Gómez, Manuel Perea

**Affiliations:** 1 Department of Psychology, Faculty of Science and Technology, Bournemouth University, Talbot Campus, Fern Barrow, Poole, Dorset BH12 5BB UK; 2 Universidad Nebrija, Madrid, Spain; 3 DePaul University, Chicago, IL USA; 4 California State University San Bernardino, Palm Desert Campus, San Bernardino, CA USA; 5 Universitat de València, Valencia, Spain

**Keywords:** Masked priming, Lexical decision task, Online experiments, PsychoPy, Prime duration

## Abstract

Masked priming is one of the most important paradigms in the study of visual word recognition, but it is usually thought to require a laboratory setup with a known monitor and keyboard. To test if this technique can be safely used in an online setting, we conducted two online masked priming lexical decision task experiments using PsychoPy/PsychoJS (Peirce et al., 2019). Importantly, we also tested the role of prime exposure duration (33.3 vs. 50 ms in Experiment 1 and 16.7 vs. 33.3 ms in Experiment 2), thus allowing us to examine both across conditions and within-conditions effects. We found that our online data are indeed very similar to the masked priming data previously reported in the masked priming literature. Additionally, we found a clear effect of prime duration, with the priming effect (measured in terms of response time and accuracy) being stronger at 50 ms than 33.3 ms and no priming effect at 16.7 ms prime duration. From these results, we can conclude that modern online browser-based experimental psychophysics packages (e.g., PsychoPy) can present stimuli and collect responses on standard end user devices with enough precision. These findings provide us with confidence that masked priming can be used online, thus allowing us not only to run less time-consuming experiments, but also to reach populations that are difficult to test in a laboratory.

Masked priming (Forster & Davis, 1984) is one of the most important techniques to study the effects of orthography, phonology, morphology, and meaning in visual word recognition (see Forster, 1998; Grainger, 2008, for reviews). Priming refers to the influence of a prime stimulus (e.g., *nurse*, *horse*) on a subsequently presented stimulus that the participant has to respond to (e.g., “is *DOCTOR* a word?”). Priming effects are measured as the difference in a dependent variable (e.g., response time [RT]) between two conditions (e.g., unrelated: *horse-DOCTOR*; related: *nurse-DOCTOR*). In masked priming, the prime stimulus is presented very briefly (for less than 60 ms) and is itself preceded by a pattern mask (e.g., #####) for a much longer duration (typically 500 ms). The rationale of the procedure is to make participants unaware of the identity of the masked prime (Forster, 1998; Forster & Davis, 1984), thus minimizing the role of participants’ strategies. Indeed, masked priming experiments do not show the strategical effects that occur with visible, unmasked primes (e.g., Grossi, 2006; Perea & Rosa, 2002).

The masked priming paradigm has been used in a large number of studies over the last decades. For instance, a search of the expression “masked priming” in Google Scholar in May 2021 produced more than 10,000 hits. Nearly all masked priming experiments have been run in a laboratory setting, often using the DMDX software developed by Forster and Forster (2003). The issue we examine in the present paper is whether masked priming experiments can be conducted in an online setting without significant changes in the pattern of results. Even before the exceptional situation due to the COVID-19 pandemic in 2020-21, in which many labs around the world were closed (or reduced their activity) for many months, online data collection had shown its many advantages: 1) easy access to a much more diverse population than that accessible at the typical university research laboratory; 2) independence from laboratory space constraints, and, often, lower costs as participants only need to be compensated for their time on the experiment; 3) no time spent commuting, waiting for the experiment to start, etc. Indeed, researchers in decision-making and economics have been using online paradigms for several decades now (e.g., Birnbaum, 2000; Paolacci et al., 2010).

Cognitive psychologists have been much slower that their behavioral economics colleagues in taking up online paradigms (see Brysbaert et al., 2016; Cai et al., 2017; Dufau et al., 2011; Eerland et al., 2013; Rodd et al., 2016, for some exceptions), often due to concerns about the validity of the results. Such concerns are not limited to cognitive studies (see Aust et al., 2013), but they are exacerbated by the reliance on precise presentation times in cognitive psychology. Furthermore, these concerns are even more central in the masked priming technique, where it is critical for the onset of the mask, the prime, and the target to occur at the nominal times. For instance, presenting the masked prime for longer than intended (e.g., 83 ms or longer instead of the nominal 50 ms) could counteract the effect of the mask, making the prime consciously visible to the participant and possibly altering the processes of interest (see Zimmerman & Gomez, 2012).

There have been attempts to address these concerns. Witzel et al. (2013) developed a Web version of DMDX (webDMDX) showing promising results in a trial experiment. However, webDMDX is a self-contained Windows executable file that participants have to download and run rather than a “true” online programming script that could be run inside of a browser. A downside of this format is that participants often are understandably skeptical about downloading and running executable files from the Internet. Additionally, many participants may not have access to a Windows PC, or may be discouraged from participating by the extra work it takes to deploy the experiment on their computer. As a consequence, the use of webDMDX in masked priming experiments has been rather limited so far (see Alluhaybi & Witzel, 2020; Dubey et al., 2018, for exceptions).

Fortunately, in recent years, there have been significant improvements in how content can be presented on the World Wide Web. Most notably, the HTML5 standard now makes it possible to use JavaScript in order to draw stimuli interactively and monitor participant responses with remarkable flexibility inside the browser. Participants do not have to install any software, and the HTML5 standard is supported by a wide variety of devices, including mobile phones and tablets (Reimers & Stewart, 2015). There have been attempts to use this technology for online masked priming experiments: Crump et al. (2013) (Experiment 7) attempted a masked priming paradigm using custom written JavaScript code, but failed to replicate the masked priming effect, which may be due to problems with their implementation (unfortunately, their study does not provide many details about the JavaScript code used). Barnhoorn et al. (2015) developed a software package called *QRTEngine* designed to run experiments within the Qualtrics online survey development environment (Qualtrics, 2020) and were able to successfully find masked priming effects (Experiment 3). Unfortunately, *QRTEngine* was only maintained for a few years and is now defunct, which is an issue with many custom-developed solutions.

Nowadays, there a variety of well-maintained software packages taking advantage of the HTML5 capabilities to present experimental stimuli and collect data, both commercial, such as Gorilla (Anwyl-Irvine, Massonni, et al., 2020b) or Testable (Rezlescu et al., 2020), and open-source such as jsPsych (de Leeuw, 2015) or PsychoJS (the JavaScript version of PsychoPy 3, Peirce et al., 2019). In addition, online setups allow researchers to target any individual with an Internet connection as a participant, from very different countries and backgrounds. Indeed, various online platforms (e.g., Prolific, Amazon Mechanical Turk) offer the possibility of recruiting participants for on-line experiments based on various specific characteristics set up by the experimenters regardless of their location (e.g., native French speakers, not older than 30 years old, not currently in college).

Of course, despite the technological advances, many cognitive psychologists still have concerns about timing and measurement precision. While JavaScript-based experiments run on the participants’ devices and thereby avoid any lag due to connection issues (e.g., to avoid delays, all stimuli are usually downloaded before the start of the experiment), experimenters have little control over which devices the experiment are run on beyond the option of explicitly preventing the experiment to run on specific device types such as mobile devices. Moreover, experimenters have no control at all over what other applications are running on the device, screen size and resolution, viewing distance, properties of the keyboard/touchscreen, etc., as all of these are determined by the device or the participants’ preferences. As Reimers and Stewart (2015) pointed out, there are two ways of testing whether timing and response issues are problematic: (1) comparing a Web-based experiment directly with an established lab-based version by measuring presentation timings (using a photodiode) and response timings on various device configurations and (2) attempting to replicate existing lab-based findings using a Web-based paradigm. If the results of the Web-based study are comparable to previous lab-based results, this suggests that, whatever the deviations in stimulus and response timing are, they are not severe enough to affect the overall findings in the paradigm in question.

The first approach has the advantage that differences in presentation timings can be objectively recorded and evaluated. A very thorough recent example of this approach is the “timing mega-study” by Bridges et al. (2020), who compared the timing in experiments run in lab-based setups with the timing in online packages run in different browsers. A very similar study by Anwyl-Irvine, Dalmaijer, et al. (2020a) compares only online packages and browsers with regard to timing. Overall, Bridges et al. (2020) found that online packages were capable of presenting visual stimuli with reasonable precision, although the lab-based packages were slightly better in this regard. This first approach is important in order to establish that a certain level of precision and accuracy can be achieved at all. If this is not possible, there is no point in moving forward to the second approach and replicating specific paradigms. However, it is of course impossible to test every possible device and configuration that participants might use. On the other hand, some of the differences in precision and accuracy between setups that can be observed using a photodiode may be too small to have an influence on actual participant performance. Therefore, we consider replication of previous key lab-based effects a more important test of online paradigms than photodiode measurements. Based on the results by Bridges et al. (2020) and Anwyl-Irvine, Dalmaijer, et al. (2020a), modern JavaScript-based stimulus presentation systems are capable of sufficiently fast and precise stimulus presentation. To establish whether masked priming studies can be successfully run online, the next step is to follow the second approach and implement the masked priming paradigm online and test whether results obtained via in-lab studies can be replicated, which is at the heart of the present study. Importantly, we will do so using PsychoPy/PsychoJS (Peirce et al., 2019), as it showed high precision and accuracy across the great majority of platforms (Anwyl-Irvine, Dalmaijer, et al., 2020a; Bridges et al., 2020), in addition to being open-source software.

Specifically, in this study we were interested in whether we could replicate and extend a key phenomenon in laboratory masked priming lexical decision using an online setup: masked identity priming is commonly described as a savings effect. As first suggested by Forster (1998), for a masked identity prime, “the lexical entry is already in the process of being opened, and hence the evaluation of this entry begins sooner,” whereas for an unrelated prime, “the entry for the target word would be closed down (since it fails to match the prime), and no savings would occur” (p. 213). Thus, according to the savings account, a target word like *DOCTOR* would enjoy an encoding advantage when preceded by an identity prime such as *doctor* than when preceded by an unrelated prime such as *pencil* (i.e., a head-start). One implication of such benefit is that the RT distributions of the unrelated and identity pairs should reflect a shift rather than a change in shape. Furthermore, this shift should be approximately similar in magnitude to the prime-target stimulus-onset asynchrony (SOA). Empirical evidence supporting this view has been obtained in several studies not only with skilled adults but also with developing readers (e.g., Gomez et al., 2013; Gomez & Perea, 2020; Taikh & Lupker, 2020; Yang et al., 2021). Gomez et al. (2013) proposed an implementation of this hypothesis within the diffusion model (Ratcliff et al., 2004). This implementation proposes that, when making a two-choice decision, the resultant RT can be explained as the sum of non-decision parameters, which are the encoding time and response execution ($T_{er}$) and decision parameters, which refer to the process of accumulation of information until a decision criteria is reached. Importantly, in the decision process, the information gathered from the stimulus can vary in noise, depending on its quality, which modifies the rate at which information is accumulated (i.e., the *drift rate*). With regards to RTs from masked priming tasks, Gomez et al. (2013) found that the difference between identity and unrelated conditions could be accounted for by a change in the $T_{er}$ parameter, while there were no differences across conditions in the parameter that corresponds to the quality of evidence gathered (i.e., drift rate)—note that changes in drift rate would necessarily produce a more skewed RT distribution in the slower, unrelated condition. The same was found by Gomez and Perea (2020) for developing readers.

Critically, the above pattern is specific to *masked* priming. In fact, the most common pattern of results in latency-based tasks is that conditions that produce longer latencies will also produce larger variance. This pattern is evident in priming as well—when primes are visible (i.e., unmasked priming), identity priming effects are stronger in the upper quantiles of the RT distribution than in the lower quantiles (i.e., a change in shape rather than a shift in RT distributions; see Gomez et al., 2013). Fits from the diffusion model show that this result corresponds to changes in both the $T_{er}$ parameter and the drift rate. Hence, when the prime is visible, it does influence the quality of the information accumulated of the target word, unlike in masked priming. Clearly, this dissociation between masked and unmasked priming reflects qualitative differences in the way primes affect the processing of the target: purely encoding in masked priming (with an expected effect close to the prime duration) vs. both encoding and information quality in unmasked priming.

In the present paper, we took advantage of the above marker to examine whether online masked priming studies follow the same pattern as in-lab masked priming studies. Specifically, we manipulated prime exposure duration in identity vs. unrelated primes: 33.3 vs. 50 ms in Experiment 1, and 16.7 vs. 33.3 ms in Experiment 2—note that targets were presented immediately after the primes (i.e., prime exposure duration was equal to the prime-target SOA). The rationale of Experiment 1 is that if the actual exposure duration of the primes is the nominal exposure duration, then we would expect the typical shift between the identity and unrelated response time distributions, which according to the savings hypothesis (Forster, 1998) would be greater for 50 ms than for 33.3-ms exposure duration (i.e., the head-start would be greater for 50 ms identity primes than for 33.3 ms identity primes). This outcome would indicate that the on-line masked priming studies reproduce a characteristic signature of laboratory masked priming studies. Alternatively, if the online presentation conditions lead to a greater actual exposure duration on the participant’s device compared to that specified in our experiment (e.g., if prime durations were, on average, 20 ms longer than intended), then the 50 ms primes may no longer be adequately masked, but may rather be consciously perceived. If this is the case, the prime could affect not only the encoding, but also core decision processes (i.e., the drift rate), which would be reflected as a stronger priming effect in the higher quantiles of the distribution (i.e., the two RT distributions would have a different shape). In this scenario, one should be very cautious when running online masked priming experiments—at least with the typical software and hardware currently available.

To further constrain the research questions, Experiment 2 was designed to be analogous to Experiment 1, except for replacing the 50-ms prime exposure duration with a very short prime exposure duration, namely, 16.7 ms. Similar extremely brief prime exposure durations have shown very weak masked priming lexical decision experiments in a laboratory setting: less than 5 ms for a prime duration of 14 ms (Ziegler et al., 2000) and less than 9 ms for a prime duration of 20 ms (Tzur & Frost, 2007)—in the Tzur and Frost (2007) experiment, this difference increased to 16.7 ms when using a very high level of contrast in the computer screen. Thus, if the size of the priming effect is roughly similar to the prime exposure duration, we would expect a much larger priming effect at the 33.3 ms prime exposure duration than at the 16.7 ms prime exposure duration. If we do observe a large effect at the 16.7 ms prime exposure duration (e.g., above 20-25 ms), this would suggest, again, that there is a qualitative difference between using the masked priming technique in the laboratory and in online experiments. Keep in mind that we are using a software that has very good control over the exposure duration (Anwyl-Irvine, Dalmaijer, et al., 2020a; Bridges et al., 2020). Figure 1 displays the link between all these verbal hypotheses, the process model that corresponds to each of them, and their predictions in the RT distributions.

All in all, replicating the results observed in controlled laboratory masked priming studies would be an important step in establishing the validity of online masked priming tasks, even in a scenario with no control over many variables (e.g., the devices in which the experiment is run, the level of contrast of the computer screen, or the additional applications that are running in the background of said device), as has been done with other paradigms that also require a high control of the presentation timing and location of the stimuli (e.g., Parker et al., 2021). This would open the possibility for masked priming researchers to collect large samples from diverse populations via online crowdsourcing methods, as has been done previously using other paradigms (e.g., Aguasvivas et al., 2020; Brysbaert et al., 2021; Brysbaert et al., 2016; Mandera et al., 2020; Ratcliff & Hendrickson, 2021). So far, this has only been possible in many-labs studies depending on the collaboration of many researchers in different countries (e.g., Adelman et al., 2014). Importantly, the present experiments go beyond an online replication: they allow us to test masked priming effects not only across conditions (identity vs. unrelated) but also within conditions (the effect of identity primes, or unrelated primes, across two prime durations) (see Jacobs et al., 1995; Ziegler et al., 2000, for advantages of within-condition comparisons). Thus, the within-subject manipulation of prime exposure duration in both experiments allows us to obtain a comprehensive picture of masked identity priming effects–also including the examination of potential inhibitory effects of unrelated primes.

**Fig. 1:**
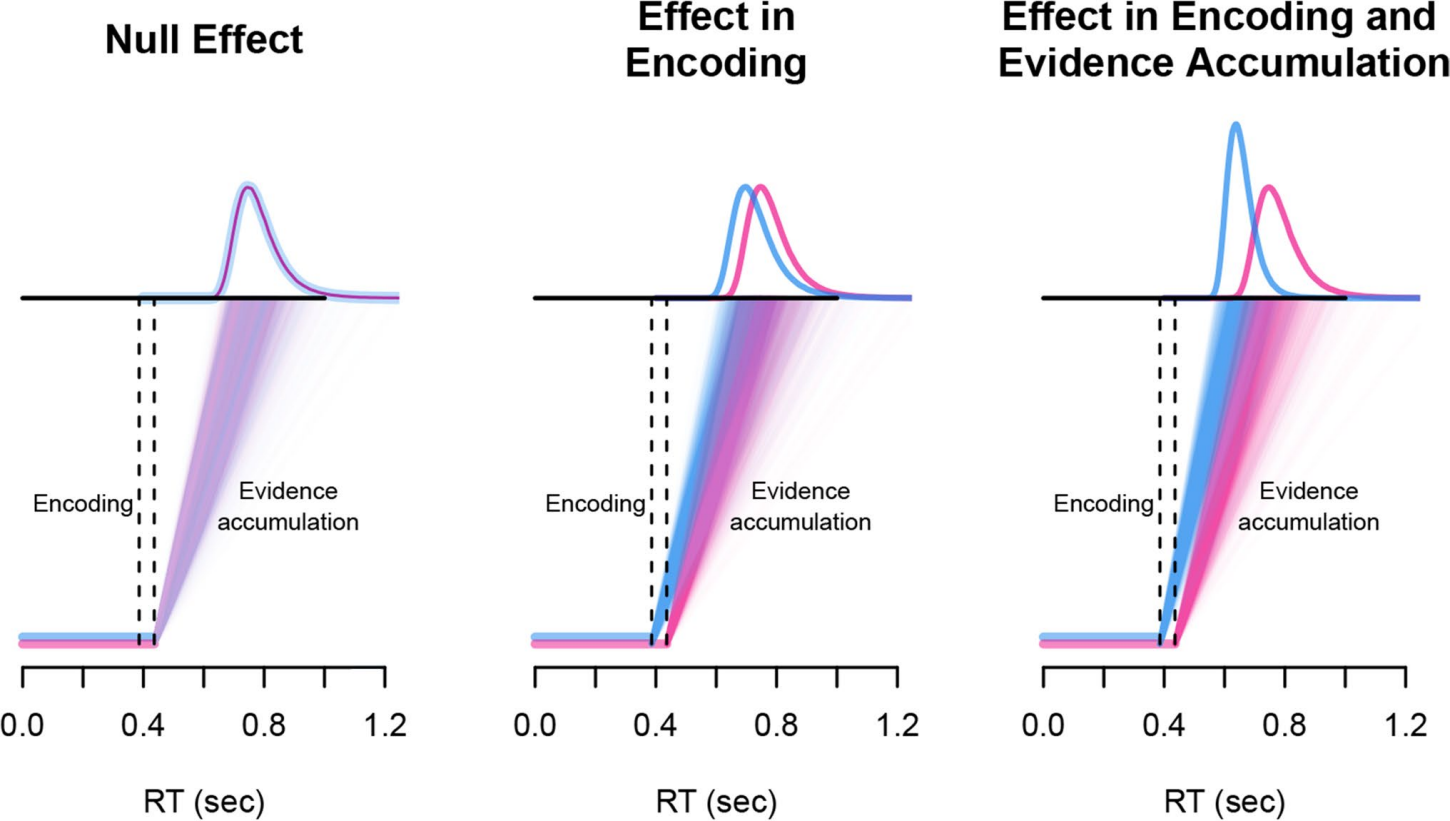
Mapping from hypotheses to data via evidence accumulation models. The *bottom part* of each graph represents sample paths, and the *top part* shows the cumulative density functions. From *left to right*: *Null Effect* - there are no differences between the conditions; *Effect in Encoding* - the difference between the conditions is on the encoding time, hence the RT distributions are shifted; and *Effect of Encoding and Evidence Accumulation* - the differences between the conditions is on the rate of evidence accumulation, thus increasing the effect size for longer RTs (right-tail of the distribution)

## Experiment 1

In the first experiment, we tested whether we could observe reliable effects of masked priming at prime durations of 33.3 and 50 ms (roughly corresponding to two and three frames, respectively, at a refresh rate of 60 Hz). We report here how we determined our sample size, all data exclusions (if any), all manipulations, and all measures in the two experiments.

### Method

The pre-registration form for Experiment 1 can be found at https://osf.io/v97bp, and the materials, data files, and R scripts can be found at https://osf.io/57rzq/.

#### Participants

Participants were recruited through Prolific (www.prolific.co, 2021). The experiment was accessed by 101 participants. Out of these, 89 provided experimental data. Two of these participants did not complete the experiment. A further ten were excluded because of low accuracy (less than .8, which was the pre-registered criterion). In the end, we analyzed the data from 77 participants (36 female), aged from 18 to 71 (mean age 31.14). All of these participants indicated that English was their first language in the Prolific screening questions. Based on their IP addresses, 47 participants were based in the UK, 23 were based in the US, three participants were based in Canada, and two participants were based in Ireland. Two participants could not be localized in this way. All participants were naïve to the purpose of the experiment and received £1.25 for their participation (corresponding to £5/h). Participants could use either a desktop/laptop computer or a mobile device. Because of a technical display issue with PsychoJS and the Safari browser, participants who tried to access the experiment using that browser, including all participants on iOS devices, were advised to change browser or device and restart the experiment.

##### Rationale for sample size and stopping rule

Brysbaert and Stevens (2018) recommended that masked priming experiments should have at least 1600 observations per condition. In order to account for potentially smaller effect sizes in an online experiment, we set a target of 3000 observations per condition (12,000 observations total), which corresponds to a minimum of 50 participants given that there were 240 word stimuli in the experiment (see below). Our stopping rule was to keep collecting data until 3000 valid observations were found. This goal was met and exceeded in our initial data collection with a budget of £200.

#### Materials

We selected 240 six-letter English words from the English Lexicon Project (Balota et al., 2007). The mean Zipf frequency based on the HAL corpus (Lund & Burgess, 1996) was 3.8 (range 1.9–5.5). The mean OLD20 (Yarkoni et al., 2008) was 2.1 (range 1.4–3). We also selected 240 matched, orthographically legal six-letter nonwords. For each target, we created an identical prime (e.g., *region — REGION* and *fainch — FAINCH*) and an unrelated prime consisting of another word from the list (e.g., *launch — REGION* and *miluer — FAINCH*)^1^. Unrelated primes were paired with the targets by rearranging the order of identity primes (controlling for neighbors; see Perea et al., 2018). Appendix 5 contains a list of the target items, and all counterbalanced lists can be found in the online repository.

#### Procedure

Participants were able to sign up for the experiment on the Prolific website. Upon signing up, they were redirected to the participant agreement form on the Qualtrics online survey development environment (Qualtrics, 2020). After indicating their agreement to participate, participants were forwarded to Pavlovia (2020), where the actual lexical decision task was implemented in PsychoJS. In the experiment, all stimuli were presented in the center of the screen in black Courier New font on a white background. As we do not know the exact dimensions of each participant’s screen, all stimulus sizes and positions were defined in PsychoPy’s “height” units, with the bottom left of a 16:10 aspect ratio screen being represented as (-.8, -.5) and the top right being (.8, .5). The height for all text stimuli was 0.1 units. Each trial began with a six-character pattern mask (*######*) set to be presented for 500 ms, followed first by the lowercase prime (e.g. *region*) set to be presented for either 33.3 or 50 ms, and then by the uppercase target (e.g. *REGION*). Participants were instructed to respond to the target stimulus as quickly as possible by pressing the “Z” key of their keyboard (if their device had one) if the target was not a valid English word or the “M” key if the target was a valid English word. Participants on a device without a keyboard were instructed to respond by touching one of two rectangular touch areas labeled “Z = Non-word” (presented at -0.4, -0.3) and “M = Word” (presented at 0.4, -0.3). The touch areas each had a width of 0.4 and a height of 0.2 and were presented in white with a black outline. If participants did not respond within 2 s of the target onset, a “Too slow!” feedback message was shown for 500 ms and the trial ended. The experimental trials were preceded by 16 practice trials during which participants also received feedback on the accuracy of their responses. No feedback apart from the trial timeout feedback was given during the experimental trials. Every 120 trials, participants were asked to take a short break before continuing the experiment. After completing the experiment, participants were redirected to a debriefing form on Qualtrics and from there back to Prolific in order to receive their participation payment.

#### Data analysis

We analyzed the data by fitting Bayesian linear and generalized linear mixed models, using the *brms* package (Bürkner, 2017, 2018) in R (R Core Team, 2021)^2^. We only analyzed trials where the target stimulus was a word. For the response time (RT) analysis, we excluded trials with RTs lower than 250 ms or higher than 1800 ms as well as incorrect responses (5.84 % of trials). For the accuracy analysis, we only excluded trials with RTs lower than 250 ms or higher than 1800 ms (0.36 % of trials). For both RTs and accuracy, we fitted a model with priming condition (unrelated vs. identical) and prime duration (33.3 ms vs. 50 ms) as well as their interaction as the fixed effects. For the discrete predictors, we used contrasts as follows: For priming condition, identical was coded as -0.5 and unrelated was coded as 0.5. For priming duration, 33.3 ms was coded as -0.5 and 50 ms was coded as 0.5. We used the maximal random effects structure possible, with random intercepts and slopes for condition, prime duration, and the interaction for participants and items. We used the ex-Gaussian distribution to model response times, with both the mean of the Gaussian component $\mu$ and the scale parameter of the exponential component $\beta$ (equaling the inverse of the rate parameter $\lambda$) being allowed to vary between conditions. To model response accuracy, we used the Bernoulli distribution with a logit link. We used the default priors suggested by *brms* except for the coefficients for the fixed effects, for which we applied weakly informative priors of $\beta \sim N(0,100)$ in order to rule out improbably large effect sizes. Each model was fitted using four chains with 5000 iterations each with 1000 warmup iterations (10,000 iterations with 2000 warm-up iterations for the accuracy models). We consider an effect as credible if the 95% credible interval (CrI) estimated from the posterior distribution does not contain zero. In addition, to better visualize the distributional features of the latency data, we computed the delta plots for both priming and prime duration effects.

### Results

Descriptive statistics for response times and accuracy for both words and nonwords in the experimental conditions are reported in Table 1 (although note that we only analyzed the word trials).

**Table 1:** Mean, Median, standard deviation (SD), range of correct RTs (in ms) and accuracy (proportion of all responses) in Experiment 1 for each condition

Stimulus Type	Prime Duration	Relatedness	Mean	Median	SD	Min	Max	Accuracy
Nonword	33.3 ms	Identity	700	665	187	263	1,776	0.91
Nonword	33.3 ms	Unrelated	702	664	188	256	1,770	0.92
Nonword	50 ms	Identity	700	658	189	256	1,760	0.91
Nonword	50 ms	Unrelated	699	657	185	251	1,796	0.93
Word	33.3 ms	Identity	630	599	164	254	1,796	0.94
Word	33.3 ms	Unrelated	652	619	166	254	1,783	0.93
Word	50 ms	Identity	608	575	161	265	1,784	0.96
Word	50 ms	Unrelated	649	620	158	294	1,710	0.93

#### Response times

Table 2 shows the mean, standard error, lower and upper bounds of the 95% CrI of the estimate of each fixed effect in the RT model, as well as the $\hat{R}$ for each estimate, which indicate that the model was fitted successfully as they are all close to 1.

The RT model indicates that the mean of the Gaussian component $\mu$ was higher in the unrelated condition than the identical (*b* = 31.55, 95% CrI [27.16, 35.92]), and higher in the 33.3 ms prime duration condition than in the 50 ms prime duration condition (*b* = -10.96, 95% CrI [-14.83, -7.11]). The interaction term indicates that the priming effect was stronger in the 50 ms condition than the 33.3 ms condition (*b* = 19.31, 95% CrI [11.39, 27.09]). The shape parameter of the exponential component $\beta$ was affected very little by prime relatedness (*b* = 0.03, 95% CrI [-0.01, 0.07]). However, the 50-ms prime exposure duration seems to be associated with a slightly lower $\beta$, i.e., a slightly weaker right skew of the distribution, than the 33.3-ms condition (*b* = -0.07, 95% CrI [-0.12, -0.03]). On the other hand, there did not seem to be an interactive effect of prime relatedness and prime exposure time on the shape of the distribution (*b* = 0.03, 95% CrI [-0.05, 0.12]).

**Table 2:** Posterior mean, standard error (SE), 95% CrI and $\hat{R}$ for the fixed effects of the model fitted for correct word RTs in Experiment 1

Parameter	mean	SE	lower bound	upper bound	$\hat{R}$
Intercept ($\mu$)	633.707	8.815	617.142	651.175	1.006
Intercept ($\beta$)	4.686	0.042	4.601	4.768	1.001
Relatedness ($\mu$)	31.551	2.228	27.162	35.915	1.000
Prime duration ($\mu$)	-10.959	1.947	-14.825	-7.105	1.000
Relatedness: Prime duration ($\mu$)	19.306	4.026	11.388	27.094	1.000
Relatedness ($\beta$)	0.031	0.022	-0.011	0.073	1.000
Prime duration ($\beta$)	-0.074	0.024	-0.120	-0.028	1.000
Relatedness: Prime duration ($\beta$)	0.031	0.042	-0.051	0.115	1.000

$\beta$ is the scale parameter (the inverse of the rate parameter $\lambda$) of the ex-Gaussian distribution

**Fig. 2:**
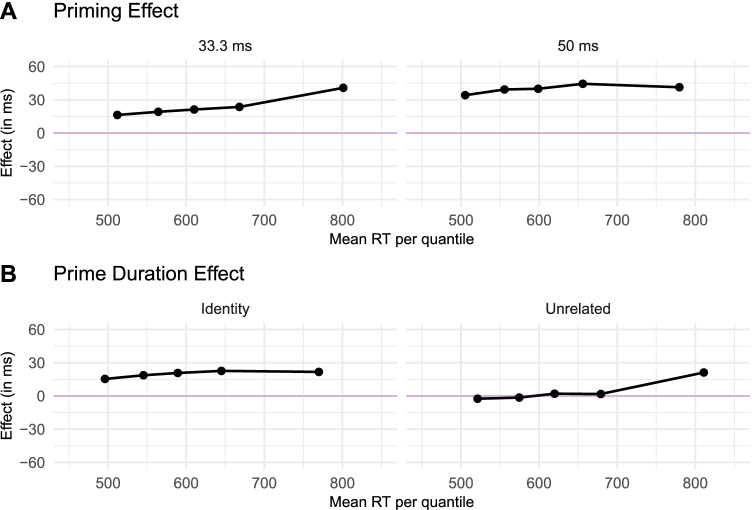
Delta plots depicting the magnitude of the effect over time in Experiment 1. Each *dot* represents the mean RT at the .1, .3, .5, .7 and .9 quantiles. *Panel A)* Difference in RT between unrelated and related trials for the 33.3-ms prime duration (left) and the 50-ms prime duration (*right*). *Panel B)* Difference in RT between 50-ms and 33.3-ms prime duration trials for the identity (*right*) and the unrelated (*left*) conditions

These results can be visualized in the delta plots depicted in Figure 2. Delta plots are residual quantile plots that show the distributional differences between conditions (see De Jong et al., 1994). As can be seen in Panel A, there is an identity priming effect (computed as the difference in response times between responses to the unrelated condition and responses to the identity condition) of a parallel magnitude across all quantiles for both the 33.3-ms and the 50-ms prime durations, and the effect size is greater for the 50-ms prime duration condition than for the 33.3-ms one. We also found a slight increase of priming effects for the longest responses (quantile .9) in the 33.3-ms prime duration condition. This apparent anomaly can be understood better by looking at Panel B of the same figure. In this panel, we show the size of the prime duration effect (computed as the difference in response times between responses to the 50-ms condition and responses to the 33.3-ms condition) across RT quantiles. While the identity condition produced a shift in the distributions of response times (i.e., the difference between conditions is constant across quantiles), the unrelated condition yielded virtually the same response times in the 33.3- and 50-ms conditions, except for a small increase at the 50-ms prime duration for the very long responses (i.e., .9 quantile)—we prefer not to over-interpret this latter finding with the slowest responses, as it did not appear in Experiment 2.

#### Accuracy

Table 3 shows the mean, standard error, lower and upper bounds of the 95% CrI of the estimate of each fixed effect in the accuracy model, as well as the $\hat{R}$ for each estimate, which indicate that the model was fitted successfully as they are all close to 1.

The accuracy model indicates that participants were less likely to produce a correct response in the unrelated condition than the identical condition (*b* = -0.50, 95% CrI [-0.72, -0.31]). The mean of the posterior distribution for prime duration suggests that accuracy was slightly lower in the 33.3-ms prime duration condition than in the 50-ms prime duration condition, but as the CrI included 0, this is not credible (*b* = 0.19, 95% CrI [-0.02, 0.42]). The interaction term indicates that the effect of priming condition on response accuracy (with the identical condition leading to higher accuracy) was stronger in the 50-ms condition than the 33.3-ms condition (*b* = -0.53, 95% CrI [-0.94, -0.14]).

**Table 3:** Posterior mean, standard error (SE), 95% CrI and $\hat{R}$ for the fixed effects of the model fitted for response accuracy on word trials in Experiment 1.

Parameter	mean	SE	lower bound	upper bound	$\hat{R}$
Intercept	3.458	0.122	3.224	3.705	1.000
Relatedness	-0.503	0.105	-0.720	-0.305	1.000
Prime Duration	0.192	0.110	-0.016	0.417	1.000
Relatedness: Prime Duration	-0.530	0.206	-0.944	-0.138	1.000

### Discussion

The results from Experiment 1 reveal that we were able to replicate benchmark masked priming effects using an online experiment. The size and shape of the effect is similar to that observed in previous studies (e.g., Gomez et al., 2013; Perea et al., 2018; Taikh & Lupker, 2020; Yang et al., 2021). In addition, we also saw a clear difference between the 33.3-ms prime duration and the 50-ms prime duration, indicating that the experiment can reliably implement timing differences of up to one frame across a variety of participant devices. While the shape of the distribution changed slightly between the 33.3-ms and the 50-ms prime exposure durations, we did not observe an effect of the prime relatedness condition on the shape parameter $\beta$ of the exponential distribution, suggesting, according to the savings hypothesis by Forster (1998), that only encoding processes were affected by the relatedness manipulation. Moreover, as can be observed in Figure 2, the effect magnitude was close to the stimulus-onset asynchrony (see also Perea et al., 2018).

Of course, just because there was a difference between the conditions, this does not necessarily mean that the timings in the two prime duration conditions actually corresponded to the display durations set in the experiment script, just that they were different. Indeed, to better define the priming effect, one needs a baseline that serves as a reference point (i.e., an analog of the minimum “intensity” priming condition; see Jacobs et al., 1995). In order to further explore this question, we performed a second experiment in which we set the prime to be displayed for an even shorter duration. As described in the Introduction, a 16.7-ms prime exposure duration should yield a negligible priming effect (Tzur & Frost, 2007; Ziegler et al., 2000) so the pattern should be qualitatively different from the 33.3- and 50-ms durations used in Experiment 1. If it does not, this would cast doubt on the timing accuracy in online experiments. In this experiment, we also include the 33.3-ms prime duration condition to not only have a better scheme to compare the two experiments, but also to be able to test the within-condition effects (Jacobs et al., 1995)—assuming the 16.7-ms prime duration serves as a baseline.

## Experiment 2

In the second experiment, we tested whether we could observe reliable effects of masked priming at prime durations of 16.7 ms and 33.3 ms (roughly corresponding to one and two frames at a refresh rate of 60 Hz).

The pre-registration form for Experiment 2 can be found at [https://osf.io/957s8]. The materials, data files, and R scripts can be found at [https://osf.io/57rzq/].

### Method

#### Participants

As in Experiment 1, participants were recruited through Prolific (www.prolific.co, 2021). The experiment was accessed by 102 participants. Out of these, 87 provided experimental data. One of these participants did not complete the experiment. A further seven were excluded because of low accuracy (again, less than .8). The remaining 79 participants were aged from 18 to 69 (mean age 31.14). Of the participants, 40 identified as male, and 39 identified as female. All these participants indicated that English was their first language in the Prolific screening questions. Based on their IP addresses, 56 participants were based in the UK, 14 were based in the US, two participants were based in Canada, two participants were based in South Africa, and one participant each was based in Hungary and Ireland. Three participants could not be localized in this way. As in Experiment 1, all participants were naïve to the purpose of the experiment, and received £1.25 for their participation (corresponding to £5/h). Participants could use either a desktop/laptop computer or a mobile device. Because of a technical display issue with PsychoJS and the Safari browser, participants who tried to access the experiment using that browser, including all participants on iOS devices, were advised to change browser or device and restart the experiment.

##### Rationale for sample size and stopping rule

As in Experiment 1, our stopping rule was to keep collecting data until 3000 valid observations were collected. This goal was met and exceeded in our initial data collection with a budget of £200.

#### Materials

The materials were identical to those used in Experiment 1.

#### Procedure

The procedure was identical to Experiment 1, the only difference being that the primes were set to be displayed for either 16.7 ms or 33.3 ms.

#### Data analysis

We analyzed the data in the same way as in Experiment 1, by only analyzing trials where the target stimulus was a word. For the response time (RT) analysis, we excluded trials with RTs lower than 250 ms or higher than 1800 ms as well as those with incorrect responses (4.93 % of trials). For the accuracy analysis, we only excluded trials with RTs lower than 250 ms or higher than 1800 ms (0.15 % of trials). For priming duration, 16.7 ms was coded as -0.5 and 33.3 ms was coded as 0.5. Otherwise, the model specifications were identical to those in Experiment 1.

### Results

Descriptive statistics for RTs and accuracy in all the experimental conditions are reported in Table 4.

#### Response times

As in Experiment 1, the RT model indicates that the mean of the Gaussian component $\mu$ was higher in the unrelated condition than the identical (*b* = 8.53, 95% CrI [5.07, 12.11]). For the main effect of prime duration, the CrI contains 0, suggesting that, when averaging across the relatedness conditions, there is no strong difference between the 16.7-ms and the 33.3-ms prime duration (*b* = -3.60, 95% CrI [-7.22, -0.03]). However, the interaction term demonstrates that this is actually due to the fact that there was a strong priming effect in the 33.3-ms condition, but only a negligible effect in the 16.7-ms condition (*b* = 15.17, 95% CrI [8.52, 21.73]). The shape parameter of the exponential component $\beta$ was affected very little by prime relatedness (*b* = 0.03, 95% CrI [-0.02, 0.07]), prime exposure duration (*b* = 0.01, 95% CrI [-0.03, 0.05]), or their interaction (*b* = -0.03, 95% CrI [-0.12, 0.05]), suggesting that the shape of the RT distribution was not affected by the manipulations in Experiment 2.

Table 5 shows the mean, standard error, lower and upper bounds of the 95% CrI of the estimate of each fixed effect in the RT model, as well as the $\hat{R}$ for each estimate, which indicate that the model was fitted successfully as they are all close to 1.

**Table 4:** Mean, median, standard deviation (SD), and range of correct RTs (in ms) and accuracy (proportion of all responses) in Experiment 2 for all conditions

Stimulus type	Prime Duration	Relatedness	Mean	Median	SD	Min	Max	Accuracy
Nonword	16.7 ms	Identity	719	680	185	330	1,787	0.93
Nonword	16.7 ms	Unrelated	717	677	183	284	1,799	0.94
Nonword	33.3 ms	Identity	717	668	193	351	1,787	0.94
Nonword	33.3 ms	Unrelated	715	672	188	281	1,789	0.94
Word	16.7 ms	Identity	659	625	160	311	1,785	0.94
Word	16.7 ms	Unrelated	661	623	165	308	1,796	0.95
Word	33.3 ms	Identity	649	618	154	280	1,657	0.96
Word	33.3 ms	Unrelated	667	633	162	269	1,701	0.94

**Table 5:** $\beta$ is the scale parameter (the inverse of the rate parameter $\lambda$) of the ex-Gaussian distribution

Parameter	mean	SE	lower bound	upper bound	$\hat{R}$
Intercept ($\mu$)	656.975	9.036	639.056	674.711	1.003
Intercept ($\beta$)	4.658	0.043	4.573	4.741	1.003
Relatedness ($\mu$)	8.529	1.795	5.065	12.106	1.000
Prime duration ($\mu$)	-3.602	1.813	-7.216	-0.033	1.000
Relatedness: Prime duration ($\mu$)	15.166	3.390	8.517	21.727	1.000
Relatedness ($\beta$)	0.028	0.023	-0.016	0.073	1.000
Prime Duration ($\beta$)	0.011	0.021	-0.031	0.053	1.000
Relatedness: Prime duration ($\beta$)	-0.034	0.043	-0.119	0.049	1.000

**Fig. 3:**
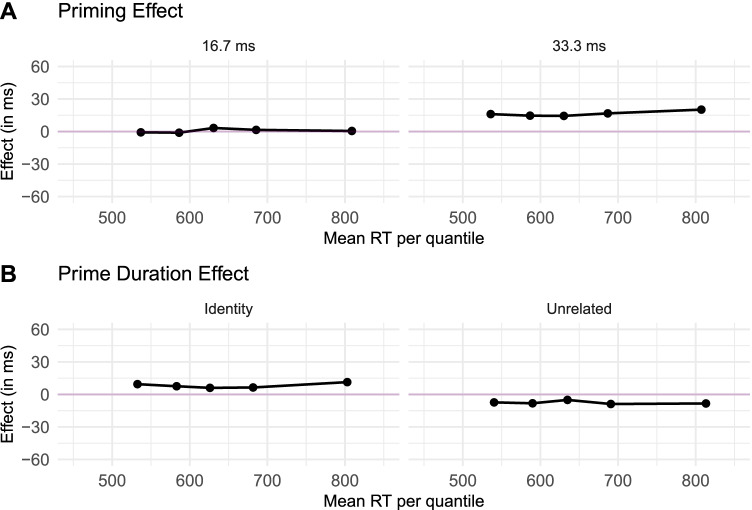
Delta plots depicting the magnitude of the effect over time in Experiment 2

We also created delta plots of the data from Experiment 2 (see Figure 3). Panel A shows there is no priming effect for the 16.7-ms condition, in contrast with a consistent effect across quantiles for the 33.3-ms condition (parallel to the one found in Experiment 1). Interestingly, when examining the delta plots for the prime duration effect, we can observe a slight facilitation for identity primes, together with a slight hindering for unrelated primes.

#### Accuracy

The accuracy model indicates that participants were less likely to produce a correct response in the unrelated condition than the identical condition (*b* = -0.22, 95% CrI [-0.43, 0.00]). The mean of the posterior distribution for prime duration suggests that accuracy was was slightly lower in the 16.7-ms prime duration condition than in the 33.3-ms prime duration condition, but as the CrI included 0, this is not credible (*b* = -0.05, 95% CrI [-0.31, 0.18]). The interaction term indicates that the expected effect of priming condition on response accuracy (with the identical condition leading to higher accuracy) only present in the 33.3-ms condition, and reversed in the 16.7-ms condition (*b* = -0.71, 95% CrI [-1.16, -0.30]), although the effect in the 16.7-ms condition was very weak. Table 6 shows the mean, standard error, lower and upper bounds of the 95% CrI of the estimate of each fixed effect in the accuracy model, as well as the $\hat{R}$ for each estimate, indicating that the model was fitted successfully as they are all close to 1.

**Table 6:** Posterior mean, standard error (SE), 95% CrI and $\hat{R}$ for fixed effects of the model fitted for response accuracy on word trials in Experiment 2

Parameter	mean	SE	lower bound	upper bound	$\hat{R}$
Intercept	3.734	0.135	3.476	4.007	1.000
Relatedness	-0.217	0.109	-0.434	-0.004	1.000
Prime Duration	-0.055	0.126	-0.306	0.184	1.000
Relatedness: Prime Duration	-0.710	0.218	-1.157	-0.305	1.000

### Discussion

In Experiment 2, we found the pattern we expected from previous research: In the mean $\mu$ of the Gaussian component of the ex-Gaussian distribution, we observed a robust identity priming effect in response time and accuracy in the 33.3-ms prime duration, but a very weak effect in the 16.7-ms prime duration (Tzur & Frost, 2007; Ziegler et al., 2000). This outcome suggests that the timing in our online experiments was likely to be quite close to the timing set in the experiment script. In addition, we did not observe an effect of the prime relatedness on the shape parameter $\beta$ of the exponential component (i.e., the effect corresponded to a shift of the RT distributions, as shown in in-lab studies; e.g., Taikh & Lupker, 2020; Yang et al., 2021). Moreover, the magnitude of the priming effect at the 33.3-ms prime exposure duration was close to the magnitude of the prime-target stimulus-onset asynchrony, as can be seen in the delta plots in Fig. 3 (see Gomez et al., 2013; Perea et al., 2018). Hence, we replicated again the commonly observed savings effect (Forster, 1998).

Critically, because the 16.7-ms prime duration condition yielded virtually no priming effects, this very short prime exposure duration serves as a within-condition baseline that allows us to qualify the facilitative vs. inhibitory nature of masked priming effects (Jacobs et al., 1995; Ziegler et al., 2000). Specifically, when compared to the baseline, our findings at the 33.3-ms prime duration show a combination of a slight facilitatory effect for identity primes and a small inhibitory effect for unrelated primes.

## General discussion

In this study, we set out to test whether we could obtain benchmark masked priming effects both qualitatively (i.e., shift in the RT distributions) and quantitatively (i.e., effect sizes) using an online, browser-based experiment software. To that end, we conducted two online masked identity priming experiments (e.g., *region — REGION* vs. *launch — REGION*) in which we manipulated prime exposure duration (33.3 vs. 50 ms in Experiment 1; 16.7 vs. 33 ms in Experiment 2). The results of our online-based experiments replicated and extended benchmarks masked identity priming effects previous in the lab-based studies. We observed the effect sizes predicted by Forster’s (1998) savings hypothesis: Our data show a shift in the mean of the Gaussian component of the ex-Gaussian distribution, but no change in the shape parameter $\beta$ of the exponential component, suggesting that our priming manipulations affected—as intended—encoding processes, but not conscious decision-making processes. Furthermore, the size of the priming effect was not only directly influenced by the prime duration, suggesting the experimental software was able to control the display timing of the prime accurately, but also of a similar magnitude to the prime duration (within the range reported in previous studies, e.g., 35-47 ms for a 50-ms prime duration in Perea et al., 2018). Importantly, the use of a within-condition baseline revealed that the identity priming effects at the 33-ms prime exposure duration were a combination of some small facilitation from identity primes and some small inhibition from unrelated primes (see Jacobs et al., 1995, for evidence with a psychophysical experiment). Likewise, the greater identity priming effects at the 50 ms rather than at the 33.3-ms prime exposure duration were essentially due to the facilitation from the identity pairs.

While accurate display timings are expected in a laboratory-based experiment, where the equipment is known and can be measured, they are much less certain in a situation where the experiment runs on a participant’s own device, which could be any of a wide variety of consumer devices including Windows PCs, Macs, tablets, and mobile phones sold in the last decade. Similarly, unlike in-lab conditions, where the contrast of the computer screens can be measured and kept constant, there is no such a guarantee for the screens used by online participants—note that Tzur and Frost (2007) were only able to observe strong effects for short prime exposure durations when using extreme contrast values. Thus, the fact that we can observe results that very closely resemble lab results demonstrates the sophistication in modern browsers’ JavaScript performance, including browsers on mobile devices, as well as the quality of the JavaScript implementation of PsychoPy (PsychoJS).

Furthermore, our results extend previous work showing the validity of on-line studies measuring response times (e.g., Brysbaert et al., 2016; Cai et al., 2017; Dufau et al., 2011; Eerland et al., 2013; Rodd et al., 2016, to cite a few instances) to the masked priming technique. Thus, the present study opens the door to a wider use of online experiments in cognitive research, especially in reaction-time sensitive fields like word recognition. Not only does this enable researchers to continue collecting data in times of social distancing, but it also makes it possible to collect data from a larger population than previously possible. For instance, online participants can be people from different countries and/or cultures, from different age groups, bilinguals/multilinguals, even those who do not own a computer, only a smartphone. Using a JavaScript-based experiment software, anyone with a smartphone can be an experiment participant. This opens up the possibility of masked-priming crowdsourced megastudies. In general, deploying masked priming experiments online also means that data can be collected very quickly and efficiently, allowing research to progress more rapidly.

In conclusion, our results give us confidence that high-quality behavioral data using the masked priming paradigm can be collected online using JavaScript-based experiment platforms. We hope that future research takes advantage of these new methods in order to make research faster, more inclusive, and more efficient.

## Open Science Statement

Both experiments in this study were pre-registered prior to conducting the research on the Open Science Framework (OSF) prior to data collection. The registration form for Experiment 1 can be found at [https://osf.io/v97bp]. The registration form for Experiment 2 can be found at [https://osf.io/957s8]. The materials, data files, and R scripts for both experiments can be found at [https://osf.io/d2txs].

## Appendix 1: Stimuli

### 1.1 Words:

**Table:**

##	[1] GLANCE WINTER MILDEW SLEAZY BOTHER PORTAL COURSE SOCIAL CENSOR BACKUP
##	[11] STABLE PLAGUE OBJECT ABSENT RESIGN CREDIT STREAM WICKED INVOKE BEACON
##	[21] ADMIRE FACTOR PHRASE BURDEN LOUNGE TAILOR WREATH HOCKEY SEARCH MONKEY
##	[31] INFECT VERIFY SQUEAL INTAKE PHOBIA BEHALF LIQUOR SHRINK BUCKET VOYAGE
##	[41] MASCOT HINDER KIDNEY PERIOD VULGAR CASINO SELDOM MASTER MOTHER FRINGE
##	[51] EXPORT INJECT NATURE CLUMSY GRUMPY PIRATE POSTER FLOWER LUMBER FOREST
##	[61] FRIGHT BRIDGE KNIGHT TRENCH PLUNGE BUNDLE ANCHOR ABRUPT HUSTLE ETHNIC
##	[71] CRUISE ORANGE STRIVE ALMOST AMOUNT FAUCET CHARGE HUMBLE PATRON BREAST
##	[81] SIMPLE JOCKEY WEALTH PRAISE DOMAIN SPRINT SCREAM COMEDY SKETCH ORIENT
##	[91] WEAPON BUCKLE BUTLER JUNGLE SOCKET VANISH INSULT POWDER INSECT CONVEY
##	[101] SPRING CHAPEL TONGUE DANGER CLERGY SINGLE INCOME WALNUT SURVEY SNATCH
##	[111] HUNGRY IMPOSE POLICE RAMBLE STROKE AMBUSH CHANGE FOSTER SPLINT PENCIL
##	[121] PONDER REGION LAUNCH THRONE SYMBOL GOSPEL POLICY FUMBLE PLENTY SQUARE
##	[131] GARDEN BRIGHT SOURCE PLAQUE TROPHY EXOTIC RACKET COLUMN THEORY FAMILY
##	[141] FRIEND STIGMA RESULT MORTAL SPRAWL RODENT FILTER HUNGER PERMIT RELISH
##	[151] EXPAND STRAND PUBLIC TRAVEL THREAD IGNORE CLIENT BELONG FLUENT CARBON
##	[161] WRENCH DIRECT BOUNCE STUDIO NUMBER RANSOM SLOGAN RECKON WONDER POLITE
##	[171] INDUCE BREATH CHORUS PLACID GUITAR TUMBLE BRANCH FLIGHT SPIDER STRAIN
##	[181] THRIVE LINGER STRIKE BLOUSE NICKEL ENOUGH CASTLE SENIOR MARVEL STUPID
##	[191] QUENCH STRONG CHROME STAPLE IMPORT BLEACH FINGER MELODY DEBRIS PRINCE
##	[201] ORPHAN SPRUCE REASON SPONGE PARISH COUPLE GARLIC CUSTOM INVADE RANDOM
##	[211] MYSTIC ROCKET BEHOLD AUTHOR LIZARD CRADLE DRAGON ISLAND DETAIL VIOLET
##	[221] PASTOR CANDLE STRING MARKET INVEST STARCH DESIGN ANSWER GENIUS POUNCE
##	[231] DEPART PATROL SHOWER TURKEY FABRIC STRIFE ADVENT INFORM BASKET SILENT

### 1.2 Non-Words:

**Table:**

##	[1] KNISMA ALCOUD STRELD PRAILE FRUDGE URIVEL SCHIND TANDLY FUNTLE CAROEY
##	[11] CHRUMS LUNKER DRANCE PITROM IMPURB FRUTAN SNAPEL BAITCH LIMPOR SARIDE
##	[21] RASTOE ABOUNE DORSEX JAUNCE MINKEL RUNAMO GIRCUE SADENT BRUNGE PHRECT
##	[31] CAINKY SURDEN BOLVIS SCOUGE TONDLE BROGET WANIGH BARCET BAFENT MINGAR
##	[41] JUSTRE ABSULT KISPEL CHAITE WHIVEX HOCITE GORBIE WICTOR PADIFS CRUDIO
##	[51] NOBEST CIGNEY CRIBLE HABLIN PEBRIC JOSTED PHOTIE ACRISE LOBUSH ROBULY
##	[61] SMENCO UNWERT MUSIED EATRIC ANOUGS PIATON BLARGE STROLE BATROL MILTED
##	[71] LUPICT STAPOD RESAIT VAROSH APIGHT INLORS VENIOD POLUTH SOUDAL UNJECT
##	[81] STUNCH QUILEW DOLICA VACKEL DISTAR VARMIT PASINK PACKES ORCHET WRIMSY
##	[91] AUBRID DAMILT SORTEL FUMBLO SLEIRT YARMON PETAIN UNIGHS CELOND CURBOL
##	[101] MELATY PORLEX VERALF SHAPLE PRUDGE RELDON NYMBEL YACEUP STARBY SPLINO
##	[111] PRETCH ABLINT SWANCE INHORT GLEATH BEINCH FERTIC PIERGY STURCH YARION
##	[121] FAINCH MILUER FIREAM CLUDIE FORGLE SOUNGE KIRAFE PSETCH AMILOY DANIET
##	[131] SPROLY NERAWL PAURSE SPRINE LASTON SCREGM WORBAL REAGLY ZOMBER JOCKAL
##	[141] MIATOR SLANCH ROSAIL STEIKH THRUSE IGUADE PLENAC BROAKE YARIKE KENSOM
##	[151] GILFEW VANGUE CATRIE WATMEG DESION GHODUS REJOLS WREASH FACHOW FIATCH
##	[161] MYRTIE RANDOW AIMOSY SCRILY ABSEND SAUDIC KERIFT DUMPLE ASYLEN NAGURS
##	[171] TIESCH TRINGE CLUREY VIRLEX ZINGLY OTHNIS DERAIN FLEACK FONVER DESORY
##	[181] PAUCHE TIREAU BREALI IMPOTE GUAIRT TOSHEL SLIQUE HORNAL WRONCH SLEACT
##	[191] GEILOY RAMILY BIGENT SLIANT YORQUE IGUARE INJORE SHRILE AUTING CAVORY
##	[201] HUNIEK SARLEY AUNGRE GUESCO VEIGMA ANCUST GRONCE CRAGIN WITMER HATRUS
##	[211] THELDY ROUNCY GANOUS SPAQUX COLURY FLENGY CAMBLE BLUNDE FOURET WEAROX
##	[221] MUSTIL SYNTIC BRUATS CLUIRM FLUMNI NOUPLY SUIVER HURNIA ELOPIA TUNORY
##	[231] ORCHIN JILUER EQUATS SERBOL HIGSTA PATHEL EAROUD SUIDLE SLOUNT GUSHOP

## Appendix 2: Diffusion model accounts for the data

### 2.1 About this Appendix

The goal of this appendix is to present a brief description of how the diffusion model accounts for the data presented in the main article. While the diffusion model fits to the grouped data was part of the pre-registration plan, we believe that it is best to present such fits in this appendix as opposed to the main text to improve the readability of the article. For a full description of the experimental setting and its goals, please refer to the main text.

### 2.2 The diffusion model

The diffusion model (Ratcliff, 1978) is a cognitive process model for perceptual decisions, and it has been quite successful at accounting for lexical decision data (Ratcliff et al., 2004) and more importantly for the present work, masked priming data (Gomez et al., 2013).

The model assumes that RTs to dual choice tasks are a sum of three distinct processes: stimulus encoding, evidence accumulation, and response execution. The model makes the strong assumption that evidence accumulation is a processes distinct from the other two components, and for practical reasons, it groups response execution and encoding time in a single parameter.

The model is agnostic about the correlation between the encoding and the evidence accumulation processes; and we like to think about it as a tool to instantiate theoretical positions that can be articulated in terms of encoding, decision, and strategic processes.

### 2.3 Data

The model was fit to the grouped data (as per the pre-registration plan) of the two experiments. For each stimulus type the proportion of word and nonword responses is calculated, then for each of the two responses, the RTs at the .1, .3, .5, .7, and .9 quantiles is obtained. We repeat this process for each participants, and then all of those quantities (response proportions and RTs at quantiles) are averaged across participants. This process is also known as Vincentizing, and the averaged quantiles are referred to as vintenciles

The diffusion model predicts the cumulative probability of a response at each RT vincentile, and these model predictions are compared to the empirical proportions, then the sum of the (Observed-Predicted)2/Predicted for correct and error responses for each condition that is minimized with a general SIMPLEX minimization routine as described by Ratcliff & Tuerlinckx (2002).

### 2.4 Free and fixed parameters

In diffusion model fits, researchers can decide what parameters are free to vary across different conditions. In the present work, we implemented three versions of the model. These versions of the model varied in terms of which parameters were allowed to vary for which conditions.

In our case, we decided to examine two models as described below. For both models, the $a$ boundary separation, the $z$ starting point, the $\eta$ between trial variability in drift rate, and all other variability parameters are kept constant across all conditions.

**Model 1:** Drift rates vary as a function of lexicality and prime duration, but not from unrelated to identity primes. $T_{er}$ varied from as a function of prime duration and type and also of lexicality.

**Model 2:** The drift rates vary as a function of prime duration and type and also of lexicality. And the drift rate varied only as a function of prime type/duration but not lexicality.

In short, in Model 1, the priming effects are accounted by $T_{er}$ only, while in Model 2 they are accounted for by both drift rate and $T_{er}$.

The two models have equal number of parameters so a direct comparison in possible. For both experiments, the preferred model is Model 1 (the $T_{er}$ model). This is in agreement with the Gomez et al (2013) study using in-person testing methods.

### 2.5 Summary

Examining the parameter values for $T_{er}$ in the tables below (Table 7, 8, and 9) shows that the $T_{er}$ effect follows the duration of the prime-TARGET SOA particularly in the word items and not so much in the nonword items.

**Table 7:** $\chi ^2$ values for the two models for both experiments

Experiments	ModelTer	ModelDrift
1	39.65	80.65
2	10.21	28.65

**Table 8:** $T_{er}$ values for Experiment 1

Parameters	Experiment1
$T_{er}$ 33ms nonword ID	0.51
$T_{er}$ 33ms nonword Unrel	0.51
$T_{er}$ 50ms nonword ID	0.50
$T_{er}$ 50ms nonword Unrel	0.51
$T_{er}$ 33ms word ID	0.47
$T_{er}$ 33ms word Unrel	0.49
$T_{er}$ 50ms word ID	0.46
$T_{er}$ 50ms word Unrel	0.50

**Table 9:** $T_{er}$ values for Experiment 2

Parameters	Experiment2
$T_{er}$ 16ms nonword ID	0.54
$T_{er}$ 16ms nonword Unrel	0.54
$T_{er}$ 33ms nonword ID	0.53
$T_{er}$ 33ms nonword Unrel	0.53
$T_{er}$ 16ms word ID	0.50
$T_{er}$ 16ms word Unrel	0.50
$T_{er}$ 33ms word ID	0.49
$T_{er}$ 33ms word Unrel	0.51

This is in general agreement with the Gomez et al. (2013) original paper and Gomez and Perea’s (2020) work with developmental readers. In short, these fits confirm that masked priming effects are consistent with the idea of a head start in the encoding process when there is an identity relationship between primes and targets.

## Footnotes

1 Note that the lexical status of the unrelated prime differed depending on the target type: for word targets, the unrelated primes were words, whereas for nonword targets, they were pseudowords. However, as shown by Fernández-López et al. (2019), the lexical status of the unrelated prime does not affect lexical decision times.

2 The full list of software we used for our analyses is as follows: R (Version 4.1.1; R Core Team, 2021) and the R-packages *bayestestR* (Version 0.11.0; Makowski et al., 2019), *brms* (Version 2.16.1; Bürkner, 2017, 2018), *dplyr* (Version 1.0.7; Wickham et al., 2021c), *forcats* (Version 0.5.1; Wickham, 2021a), *ggplot2* (Version 3.3.5; Wickham, 2016), *papaja* (Version 0.1.0.9997; Aust & Barth, 2020), *patchwork* (Version 1.1.1; Pedersen, 2020), *purrr* (Version 0.3.4; Henry & Wickham, 2020), *Rcpp* (Eddelbuettel & Balamuta, 2018; Version 1.0.7; Eddelbuettel & François, 2011), *readr* (Version 2.0.2; Wickham & Hester, 2020), *readxl* (Version 1.3.1; Wickham & Bryan, 2019c), *rworldmap* (Version 1.3.6; South, 2011), *see* (Version 0.6.8; Lüdecke et al., 2020), *sp* (Version 1.4.5; Pebesma & Bivand, 2005), *stringr* (Version 1.4.0; Wickham, 2019a), *tibble* (Version 3.1.5; Müller & Wickham, 2021), *tidyr* (Version 1.1.4; Wickham, 2021b), *tidyverse* (Version 1.3.1; Wickham et al., 2019b), *tinylabels* (Version 0.2.1; Barth, 2021), and *xfun* (Version 0.27; Xie, 2021).
